# Progression of metastatic castrate-resistant prostate cancer: impact of therapeutic intervention in the post-docetaxel space

**DOI:** 10.1186/1756-8722-4-18

**Published:** 2011-04-23

**Authors:** A Oliver Sartor

**Affiliations:** 1 Departments of Medicine and Urology, Tulane University School of Medicine, 1430 Tulane Ave., SL-42, New Orleans, LA 70115, USA

## Abstract

Despite the proven success of hormonal therapy for prostate cancer using chemical or surgical castration, most patients eventually will progress to a phase of the disease that is metastatic and shows resistance to further hormonal manipulation. This has been termed metastatic castrate-resistant prostate cancer (mCRPC). Despite this designation, however, there is evidence that androgen receptor (AR)-mediated signaling and gene expression can persist in mCRPC, even in the face of castrate levels of androgen. This may be due in part to the upregulation of enzymes involved in androgen synthesis, the overexpression of AR, or the emergence of mutant ARs with promiscuous recognition of various steroidal ligands. The therapeutic options were limited and palliative in nature until trials in 2004 demonstrated that docetaxel chemotherapy could significantly improve survival. These results established first-line docetaxel as the standard of care for mCRPC. After resistance to further docetaxel therapy develops, treatment options were once again limited. Recently reported results from phase 3 trials have shown that additional therapy with the novel taxane cabazitaxel (with prednisone), or treatment with the antiandrogen abiraterone (with prednisone) could improve survival for patients with mCRPC following docetaxel therapy. Compared with mitoxantrone/prednisone, cabazitaxel/prednisone significantly improved overall survival, with a 30% reduction in rate of death, in patients with progression of mCRPC after docetaxel therapy in the TROPIC trial. Similarly, abiraterone acetate (an inhibitor of androgen biosynthesis) plus prednisone significantly decreased the rate of death by 35% compared with placebo plus prednisone in mCRPC patients progressing after prior docetaxel therapy in the COU-AA-301 trial. Results of these trials have thus established two additional treatment options for mCRPC patients in the "post-docetaxel space." In view of the continued AR-mediated signaling on mCRPC, results from additional phase 3 studies with novel antiandrogens which are directed at inhibition of the AR (e.g., MDV3100), as well as other agents, are awaited with interest and may further expand the treatment choices for this difficult-to-manage population of patients.

## Introduction

Prostate cancer is the most frequently diagnosed non-skin cancer, and the second leading cause of cancer death, in men residing in the United States [1]. It is well understood that the initial growth of prostate cancer is dependent on androgens; therefore, hormonal therapy remains a first-line treatment [2-4]. Initial responses to hormonal therapy with chemical or surgical castration are quite favorable, with rapid biochemical responses, as assessed by declines in levels of the serum marker, prostate-specific antigen (PSA) [3,5,6]. However, most patients showing an initial response to hormonal therapy for prostate cancer will progress to a castration-insensitive phase of the disease which carries a much poorer prognosis [3,4,6]. Treatment of patients with metastatic castrate-resistant prostate cancer (mCRPC) remains a significant clinical challenge.

In 2004, the results of two major phase 3 clinical trials established docetaxel as a primary chemotherapeutic option for patients with mCRPC [7,8]. Additional hormonal treatment with antiandrogens, chemotherapy, combination therapies, and immunotherapy, has been investigated for mCRPC, and recent results have offered additional options in this difficult-to-treat patient group [9,10].

In initial studies, median survival of men with mCRPC treated with chemotherapy were reported as less than 1 year [11]; more recently, survival times of approximately 22 months have been observed [12]. In this review, we examine treatment options for mCRPC, particularly for men who progress following treatment with first-line chemotherapy with docetaxel/prednisone, the current standard of care.

## Molecular aspects of CRPC

### Evidence for persistent androgen dependence

Studies have suggested, even in the presence of castrate levels of androgen, androgen levels in the prostate of men with CRPC still remain nearly equivalent of those in non-castrate patients [13]. The source of these androgens is thought to be derived from synthesis of the androgens directly in prostate cancer cells due to an upregulation of the enzymes necessary to synthesize androgens such as testosterone and dihydrotestosterone [14,15]. These findings suggest that prostate cancer that recurs despite castrate serum testosterone levels is not truly androgen-independent.

Several other mechanisms also may result in activation of the AR in prostate cancer in the face of castrate levels of androgen. These include increased AR expression through gene amplification and other mechanisms [16], mutations of the AR that can affect its ligand promiscuity, and molecular cross-talk with other signaling pathways and co-regulators that lie downstream of the AR [2,5,17].

Studies from Hu et al. [18] have shown that splice variants of the AR may be identified that encode ligand-domain deleted proteins that are constitutively activated and more abundantly expressed in CRPC than in hormone-naïve disease. Studies from Sun et al. [19] also have identified splice variants of the AR that are truncated and constitutively activated. Recent data from Watson et al. [20] suggest that expression of splice variants of the AR in CRPC actually could be dependent on hormonal therapy, such that these variants are expressed within days of castration, and diminish after androgen treatment. These androgen-independent variants of the AR are sufficient to confer castration-resistant growth to prostate cancer cells. Interestingly, however, in model systems they may be inhibited by antiandrogens targeted to the ligand-binding domain, such as MDV3100 (see below) [20]. Hypothetically this may be a consequence of inhibiting wild-type ARs, which form heterodimers with truncated splice variant ARs.

Multiple pathways may be amenable to therapeutic intervention for patients with CRPC. In view of the persistence of both AR and tissue androgens in recurrent prostate cancer, therapies that directly target the AR, or affect the persistence of androgens in prostate tissue, may be of value for patients with CRPC [13,20]. However, additional therapeutic strategies, including chemotherapy and immunotherapy, also have demonstrated benefit in CRPC, particularly in terms of the most important outcome of improved survival.

## Treatment options - then and now

Prior to 2004, there was no treatment proven to improve survival for men with mCRPC. The treatment of patients with mitoxantrone with prednisone or hydrocortisone was aimed only at alleviating pain and improving quality of life, but there was no benefit in terms of overall survival (OS) [11,21]. In 2004, however, two key trials, TAX 327 and SWOG (Southwest Oncology Group) 9916, demonstrated a benefit for docetaxel-based regimens in the treatment of men with CRPC [7]. In TAX 327, a 24% relative reduction in death for men with mCRPC was observed with a 3-weekly docetaxel with prednisone regimen (hazard ratio [HR] for death = 0.76; 95% confidence interval [CI] = 0.62-0.94), and the benefit in survival rate compared with patients receiving mitoxantrone and prednisone was significant (P = 0.009) [7]. Docetaxel also was effective in providing palliative relief, with 35% of patients reporting reduction in pain vs 22% with mitoxantrone (P = 0.01) [7]. Outcomes of TAX 327 demonstrated that chemotherapy with docetaxel was a viable option that prolonged survival for patients with mCRPC [7]; moreover, with an extended follow-up, the survival benefit of docetaxel in the TAX 327 trial has persisted [22]. In SWOG 9916, a regimen of docetaxel + estramustine was compared with mitoxantrone and prednisone [8]. In this study, the docetaxel regimen also conferred a significant survival benefit over the comparator (HR for death = 0.80; 95% CI = 0.67-0.97), and increased median survival (17.5 vs 15.6 months; P = 0.02) [8]. At present, docetaxel/prednisone remains the first-line chemotherapy of choice for patients with CRPC.

### Docetaxel combinations

Combinations of docetaxel and different drug classes, including tyrosine kinase inhibitors, antiangiogenesis agents, and immunologic agents, have been evaluated in phase 2 studies for CRPC [23]. Whereas trials of some combinations, such as GVAX and DN-101, have terminated early due to increased toxicity and poorer survival, combination trials with other agents, including aflibercept and dasatinib, are under way [23,24]. A phase 2 study (N = 60) of docetaxel, bevacizumab, thalidomide, and prednisone found 50% or greater PSA declines in 90% of patients with mCRPC and a median overall survival of 28.2 months. Toxicity of the regimen was manageable but, notably, virtually all patients developed grade 3 or 4 neutropenia [25]. Addition of bevacizumab to docetaxel did not prolong survival in a recently reported CALGB trial [12]. Thus far, phase 3 data for combination therapy with docetaxel has not produced any viable therapeutic options. Relevant phase 3 trials under way in this area include combination of docetaxel with antiangiogenesis agents such as aflibercept, bone-seeking radioisotopes such as strontium-89, inhibitors of endothelin receptors such as zibotentan (ZD4054; see below), and signal transduction/kinase inhibitors such as dasatinib.

## The post-docetaxel space

Although docetaxel produces a modest survival benefit in patients, the therapy is not curative and some patients will require treatment with additional therapies. Such is the nature of the current "post-docetaxel space," which, until recently, was devoid of a viable treatment option for CRPC patients following progression post-docetaxel. A number of treatment modalities have been proposed; those with most promising trial results, outlined in further detail below. Docetaxel retreatment has been advocated by some investigators [26], but given recent advances with newer medications, it is likely this approach will be used less often in the future. In addition, ketoconazole has substantial activity in prostate cancer both pre- and post-docetaxel [27,28]. It should also be mentioned that sunitinib, despite phase 2 supportive data [29], recently failed in a phase 3 trial.

### Cytotoxic agents

#### Satraplatin - SPARC trial

In the phase 3 SPARC trial, satraplatin, a third-generation, platinum-based chemotherapy, was used with prednisone in patients with CRPC who progressed through at least one prior chemotherapy (N = 950) [24,30]. Results for the co-primary endpoint of progression-free survival (PFS) showed that satraplatin reduced risk for progression or death by 33% compared with placebo (HR = 0.67; 95% CI = 0.57-0.77; P < 0.001). OS, however, was not significantly different between the groups (HR = 0.98; 95% CI = 0.84-1.15; P = 0.80) [30]. The drug was generally well tolerated; key adverse events (AEs) were myelosuppression and gastrointestinal disorders, which were more frequent with satraplatin.

#### Cabazitaxel - TROPIC

Cabazitaxel [10] is a novel taxane-class cytotoxic agent [31] that has shown efficacy in model system tumors that are resistant to paclitaxel and docetaxel [32,33]. In a recently published, randomized, multicenter, phase 3 trial, the efficacy and safety of cabazitaxel and prednisone were compared with those of mitoxantrone and prednisone for the treatment of mCRPC that had progressed following docetaxel-based chemotherapy [10]. A total of 755 patients were randomly assigned to treatment with cabazitaxel (N = 378) or mitoxantrone (N = 377), and the median follow-up for both treatment groups was 12.8 months [10]. Kaplan-Meier analysis demonstrated a significant benefit in OS for patients assigned to cabazitaxel, with a significant (P < 0.0001) 30% reduction in death (HR = 0.70; Table 1); the median OS was 15.1 months with cabazitaxel, compared with 12.7 months with mitoxantrone. The composite endpoint of median PFS (defined as the time between randomization and first date of PSA progression, tumor progression, pain progression, or death) also favored the cabazitaxel arm (2.8 vs 1.4 months; P < 0.0001). Tumor response (14.4% vs 4.4%; P = 0.0005) and PSA response (39.2% vs 17.8%; P = 0.0002) also significantly favored cabazitaxel, as did median time to tumor progression (8.8 vs 5.4 months; P < 0.0001) and median time to PSA progression (6.4 vs 3.1 months; P = 0.001). Pain response and time to pain progression were similar between the treatment groups [10]. Hematologic toxicities (neutropenia, leukopenia, anemia) were the predominant grade 3 or higher AEs associated with cabazitaxel in the study; the most common clinical grade 3 or higher AEs were febrile neutropenia and diarrhea. Grade 3 neuropathy was uncommon (1% for cabazitaxel). The findings of TROPIC established cabazitaxel as the first agent to prolong survival in the post-docetaxel space, with a 30% reduction in death over mitoxantrone [10]. On the basis of these data, cabazitaxel has been approved by the US Food and Drug Administration for use in patients with mCRPC who have progressed after docetaxel [34]. Results of subgroup analysis in TROPIC also should be mentioned, as these showed a benefit of cabazitaxel over mitoxantrone in patients progressing during docetaxel treatment and in those receiving more prolonged dosing with docetaxel [10]. In the TROPIC trial, the median time from last docetaxel dose to progression was less than one month; also, these patients were heavily pretreated with a median of 7 cycles of docetaxel pre-TROPIC enrollment. Still, it should be noted that cabazitaxel can be associated with substantial toxicity, and deaths within 30 days of the last dose were reported in 4.9% of patients. This toxicity was primarily related to myelosuppression, but diarrhea could also be severe. First cycle monitoring with weekly CBC is recommended and primary prophylaxis with G-CSF is recommended for men over the age 65 and for those with a poor performance status, previous episodes of febrile neutropenia, extensive prior radiation ports, poor nutritional status, or other serious comorbidities [35].

**Table 1:** Currently proven treatment options for mCRPC patients in the post-docetaxel space [9,10]

Drug (trial)	Class (comparator)	Primary outcomes vs comparator (hazard ratio [HR])	95% CIs	P value
**Abiraterone acetate + prednisone (COU-AA-301)**	Antiandrogen (prednisone/placebo)	Median OS = 14.8 vs 10.9 mo (HR = 0.65)	0.54-0.77	< 0.0001
		Median TTPP = 10.2 vs 6.6 mo (HR = 0.58)	0.46-0.73	< 0.0001
		Median rPFS = 5.6 vs 3.6 mo (HR = 0.67)	0.58-0.78	< 0.0001
		PSA response: 38% vs 10%	-	< 0.0001

**Cabazitaxel + prednisone (TROPIC)**	Chemotherapy (mitoxantrone/pred-nisone)	Median OS = 15.1 vs 12.7 mo (HR = 0.70)	0.59-0.83	< 0.0001
		Median PFS = 2.8 vs 1.4 mo (HR = 0.74)	0.64-0.86	< 0.0001
		Tumor response = 14.4% vs 4.4%	-	0.0005
		PSA response = 39.2% vs 17.8%	-	0.0002

CIs: confidence intervals; mCRPC: metastatic castrate-resistant prostate cancer; mo: months; OS: overall survival; PFS: progression-free survival; PSA: prostate-specific antigen; rPFS: radiologic PFS; TTPP: time to pain progression.

#### Abiraterone Acetate - COU-AA-301

Antiandrogen therapy is designed to further inhibit androgen-mediated signaling, which may be mediated by residual adrenal androgen in prostate tissue [4]. Interim analysis of the COU-AA-301 trial was recently reported at the 2010 European Society for Medical Oncology Congress [9]. In this trial, the safety and efficacy of abiraterone acetate (AA) with prednisone was compared with that of placebo and prednisone in men with mCRPC previously treated with docetaxel, with the primary endpoint of OS. AA is an orally administered pregnenolone analog, which further reduces androgen levels in CRPC via the inhibition of CYP17, a rate-limiting enzyme in androgen biosynthesis [36]. This drug has been shown to have activity in mCRPC with acceptable toxicity in phase 1 studies [37]. Principle side effects associated with this agent include hypertension, hypokalemia, and edema, which appear to be manageable with mineralocorticoid antagonists or low-dose corticosteroids [36,38]. In COU-AA-301, both fluid retention (30.5% vs 22.3%) and hypokalemia (17.1% vs 8.4%) were more common with AA compared with placebo, whereas grade 3 and 4 hypokalemia (3.8% vs 0.8%) and hypertension (1.3% vs 0.3%) were observed infrequently [9]. The efficacy findings of the trial (Table 1) prompted the Independent Data Monitoring Committee to recommend unblinding the trial at the time of the interim analysis and the crossover of patients from the placebo arm to AA. These results showed a significant improvement in OS, time to PSA progression, radiographic PFS, and PSA response for patients treated with AA, relative to those on placebo (Table 1) [9]. The results of COU-AA-301 confirm that targeting persistent androgen biosynthesis is a viable therapeutic option for men with progressive disease despite medical or surgical castration. A new drug application (NDA) was filed with the US regulatory authorities in December 2010, with abiraterone/prednisone currently being evaluated in a phase 3 trial in metastatic, chemotherapy-naïve CRPC patients.

#### MDV3100

As noted earlier, therapies that effectively and directly block AR activity, as opposed to suppressing residual androgen levels, may be of therapeutic value in CRPC. MDV3100 is an AR antagonist that blocks androgen binding and prevents nuclear translocation and recruitment of coactivators [24,39]; it has been shown to confer a tumor response in men with CRPC after failure of prior hormonal therapy, with 43% showing a ≥50% PSA response in a phase 1/2 study [40,41]. In another phase 1/2 study of men with CRPC without metastases, MDV3100 demonstrated antitumor activity in men both with and without prior chemotherapy exposure, validating the importance of continued AR signaling in tumor growth for men with CRPC [39]. As noted earlier, AR splice variants lacking the ligand-binding domain have been identified, and these were predicted to play an important role in the development of castration resistance in prostate cancer; surprisingly, the cells expressing these variants were found to be inhibited by MDV3100 despite the absence of a ligand-binding domain in some ARs [20]. These findings suggest that MDV3100 could partly prevent some of the androgen independence conferred by these variants in prostate cancer patients. Currently, there are two phase 3 clinical trials under way with MDV3100 for men with CRPC, one of which will examine safety and efficacy in men with metastatic chemotherapy-naïve disease (ClinicalTrials.Gov: NCT01212991), and another (ClinicalTrials.Gov: NCT00974311) examining safety and efficacy of MDV3100 in men post docetaxel therapy [39]. The post-docetaxel therapy trial recently completed accrual. Newer agents designed to block CYP17 activity such as TAK-700 are also now in phase 3 trials both pre- and post-docetaxel in metastatic CRPC. In addition, ARN-509 is a new and potent antiandrogen in clinical development.

### Vaccine immunotherapy

#### Sipuleucel-T

Sipuleucel-T is a vaccine-type immunotherapy designed to stimulate an immune response to prostate cancer cells [42]. In a small (N = 127), placebo-controlled, phase 3 study (N = 82 sipuleucel-T, N = 45 placebo), sipuleucel-T was found to confer a significant 4.5-month benefit in survival for men with mCRPC, and the treatment was generally well tolerated (prior chemotherapy was permitted if at least 6 months had elapsed, or 3 months, if the CD4+ cell count was > 400) [42,43]. These findings formed the basis for the IMPACT trial, which examined the efficacy and safety of sipuleucel-T (N = 341) or placebo (N = 171) in patients with mCRPC with asymptomatic or minimally symptomatic disease, and an expected survival of at least 6 months [42]. Prior to enrollment in the phase 3 sipuleucel-T trial, patients had to be post-chemotherapy for longer than 3 months and not have visceral metastases; in addition, these patients were required to be asymptomatic or minimally symptomatic. Sipuleucel-T treatment reduced the relative risk of death by 22% (HR = 0.78; 95% CI = 0.61-0.98; P = 0.03) and increased median survival by 4.1 months (25.8 vs 21.7). Despite the observation of survival extension, however, no effects were seen on either tumor response or time to tumor progression. The treatment was well tolerated with predominantly grade 1 and 2 infusion-related AEs such as fever and chills [42]. The findings of IMPACT demonstrated the first significant survival benefit for an immunotherapy in patients with CRPC. It should be noted, however, that the trial involved mostly patients who were docetaxel-naïve (≥85%), so the utility of this treatment in the post-docetaxel space requires further study [42].

In addition to sipuleucel-T, various other vaccination approaches are under development in prostate cancer. In a randomized phase 2 trial, PROSTVAC-VF demonstrated an improvement in survival without effects on disease progression in patients with asymptomatic or minimally symptomatic metastatic CRPC [43]. This agent uses a PSA antigen presented in the context of 3 co-stimulatory molecules (ICAM-1, BLA-7, and LFA-3) which, when taken together, demonstrate an increase in strength of the targeted immunologic response. A phase 3, 1,200-patient trial with PROSTVAC-VF is planned in the near term future. Other novel vaccine approaches under current development include transdermally administered dendritic cells pulsed with PSMA peptides and transduced with a modified CD40 which can be activated in vivo with chemically defined chemical moiety [44]. These modifications permit prolonged activation of CD40-expressing dendritic cells.

### Endothelin receptor antagonism

As noted earlier, activation of other signaling pathways may play a role in the emergence of CRPC. The interaction of endothelin-1 (ET-1) with the G-protein coupled endothelin-A (ET_A_) receptor has been implicated in carcinogenesis, and results in the triggering of several intracellular signaling pathways [45]. ET-1 and the ET_A _receptor may be involved in a number of processes in CRPC, including cell growth and survival, angiogenesis, development of bone metastases, and the nociceptive response [45,46]. ZD4054 is a potent, orally available endothelin receptor antagonist that has a high selectivity for the ET_A _receptor [45]. In phase 2 studies, ZD4054 improved survival significantly for men with CRPC and bone metastases (HR = 0.55; 95% CI = 0.41-0.73; P = 0.008) [47]. Placebo-controlled, phase 3 clinical trials were planned for ZD4054; however, interim analyses have failed to demonstrate a significant benefit on survival for patients with mCRPC [48]. Recent results also indicate that non-metastatic CRPC patients fail to benefit. Final results of the trial with docetaxel/prednisone are pending [23]. Atrasentan is another antagonist of ET_A _that has been evaluated in two placebo-controlled phase 3 studies of men with CRPC [24]. Thus far, significant effects on disease progression and survival have not been observed with this agent, but SWOG has an ongoing phase 3 trial that has completed accrual in combination with docetaxel/prednisone [24].

## Selection of therapy for CRPC

With the range of newer treatment options becoming available, it is clear there will be a need to more carefully define the most appropriate treatment for individual patients with CRPC. As the incidence of prostate cancer is disproportionately high in elderly men, consideration should be given to life expectancy issues, functional status, and the ability of a patient to tolerate potential side effects of therapies [49]. Because elderly patients also may benefit from chemotherapies to the same degree that younger patients do, we should ensure that all treatment options that prolong survival, control symptoms, reduce pain, and improve quality of life are available to those patients with good clinical status[49]. Strategies such as proteomic profiling have been used to define markers that predict docetaxel resistance in men with mCRPC, and use of such biomarkers potentially could better define which patients will experience recurrence early on docetaxel therapy and direct these patients to a more appropriate therapy [50]. Other surrogate biomarkers for prediction of clinical benefit in mCRPC include PSA, bone turnover markers, bone pain, bone scans, and circulating tumor cells [51]. The use of these surrogate biomarkers has the potential to improve patient selection strategies, and more rapidly identify agents that merit further testing in phase 3 clinical trials, as well as accelerate phase 3 testing. However, these markers will require validation for use in patients with mCRPC [51].

## Conclusions and future prospects

While initial responses to hormone-based therapies for prostate cancer are favorable, patients ultimately will progress to CRPC that displays resistance to traditional hormonal manipulation. Previous therapies for CRPC were of a palliative nature, and no proven survival benefit for a CRPC treatment was established until 2004, when docetaxel was proven to prolong survival. The use of a docetaxel-based regimen as first-line chemotherapy is now considered a standard of care for men with mCRPC. It is now clear that other treatment modalities, including immunotherapy with sipuleucel-T, are effective options for patients with asymptomatic or minimally symptomatic mCRPC. Patients in the "post-docetaxel space" have presented the greatest challenge for ongoing research over the past several years. Thus far, two agents have shown considerable activity in this setting, including cabazitaxel and AA (Table 1). The critical endpoint in the "post-docetaxel" space has been extending OS; the results of recent phase 3 trials with OS as a primary endpoint have been encouraging (Table 1). In view of the persistence of androgen signaling in mCRPC, results from additional ongoing phase 3 studies, particularly with novel therapies targeted at the AR (e.g., MDV3100), also are awaited with interest. In addition, patient selection for the particular type of therapy will be all-important, and further research is necessary to define patient characteristics and subgroups most able to benefit from each of these emergent therapies. The future will likely bring a number of possibilities for combination therapy, sequential therapy, and other treatment modalities advantageous to certain subgroups within the difficult-to-treat population of patients with mCRPC.

## List of abbreviations

AA: abiraterone acetate; AEs: adverse events; AR: androgen receptor; CI: confidence interval; ET-1: endothelin-1; ET_A_: endothelin-A; HR: hazard ratio; mCRPC: metastatic castrate-resistant prostate cancer; OS: overall survival; PFS: progression-free survival; PSA: prostate-specific antigen; SWOG: Southwest Oncology Group; ZD4054: zibotentan

## Competing interests

**Consultant**: sanofi-aventis, Medivation, Centocor, Dendreon, Celgene, Bristol-Myers Squibb

**Investigator**: sanofi-aventis, Cougar/Centocor

## Authors' contributions

OS developed and drafted the manuscript

## Author's information

OS is Laborde Professor for Cancer Research and is in the Departments of Medicine and Urology at Tulane University School of Medicine
